# Differentiating proven progressive disseminated histoplasmosis from other diagnoses in hospitalized persons with HIV and suspected progressive disseminated histoplasmosis: Findings from a clinical and demographic study in Mexico

**DOI:** 10.1371/journal.pntd.0013527

**Published:** 2025-09-17

**Authors:** Maria Dolores Niembro-Ortega, Areli Martinez-Gamboa, Antonio Olivas-Martinez, Brenda Crabtree-Ramirez, Janeth Santiago-Cruz, Andrea Rangel-Cordero, Pedro Torres-Gonzalez, Armando Gamboa-Dominguez, Victor Hugo Lozano-Fernandez, Victor Hugo Ahumada-Topete, Pedro Martinez-Ayala, Marisol Manriquez-Reyes, Juan Pablo Ramirez-Hinojosa, Patricia Rodriguez-Zulueta, Jesus Ruiz-Quiñones, Christian Hernandez-Leon, Norma Erendira Rivera-Martinez, Alberto Chaparro-Sanchez, Jaime Andrade-Villanueva, Luz Alicia Gonzalez-Hernandez, Sofia Cruz-Martinez, Oscar Flores-Barrientos, Jesus Enrique Gaytan-Martinez, Axel Cervantes Sanchez, Nancy Guadalupe Velazquez-Zavala, Maria del Rocio Reyes-Montes, Esperanza Duarte Escalante, Maria Guadalupe Frias De Leon, Jose Antonio Ramirez, Maria Lucia Taylor, Jose Sifuentes-Osornio, Alfredo Ponce de Leon

**Affiliations:** 1 Department of Infectious Diseases, Instituto Nacional de Ciencias Médicas y Nutrición “Salvador Zubirán”, Mexico City, Mexico; 2 Departmet of Medicine, Instituto Nacional de Ciencias Médicas y Nutrición “Salvador Zubirán”, Mexico City, Mexico; 3 Department of Biostatistics, University of Washington, Seattle, Washington, United States of America; 4 Department of Pathology, Instituto Nacional de Ciencias Médicas y Nutrición “Salvador Zubirán”, Mexico City, Mexico; 5 Centro de Investigación en Enfermedades Infecciosas, Instituto Nacional de Enfermedades Respiratorias “Ismael Cosio Villegas”, Mexico City, Mexico; 6 HIV Unit, Hospital Civil de Guadalajara “Fray Antonio Alcalde”, Guadalajara, Jalisco, Mexico; 7 Department of Internal Medicine, Hospital de Alta Especialidad de Veracruz, Veracruz, Veracruz, Mexico; 8 Department of Infectious Diseases, Hospital General “Dr. Manuel Gea González”, Mexico City, Mexico; 9 Intensive Care Unit, Department of Internal Medicine, Hospital “Dr. Juan Graham Casasus”, Villahermosa, Tabasco, Mexico; 10 Area of Infectious Diseases, Department of Internal Medicine, Hospital General de Puebla “Dr. Eduardo Vázquez Navarro”, Puebla, Puebla, Mexico; 11 Adult Infectious Diseases Department, Hospital Regional de Alta Especialidad de Oaxaca, HRAEO, San Bartolo Coyotepec, Oaxaca, Mexico; 12 Department of Infectious Diseases, Hospital de Infectología del Centro Médico Nacional “La Raza”, Instituto Mexicano del Seguro Social, Azcapotzalco, Mexico City, Mexico; 13 Mycology Unit, Department of Microbiology and Parasitology, Facultad de Medicina, Universidad Nacional Autónoma de México, Mexico City, Mexico; 14 General director at Instituto Nacional de Ciencias Médicas y Nutrición “Salvador Zubiran”, Mexico City, Mexico; Albert Einstein College of Medicine, UNITED STATES OF AMERICA

## Abstract

**Background:**

Progressive disseminated histoplasmosis (PDH) is a leading cause of morbidity and mortality among persons with HIV (PWH) in the Americas. Clinical presentation often overlaps with other opportunistic infections —especially tuberculosis (TB)— and sensitive diagnostics are frequently unavailable. In Mexico, epidemiological data on histoplasmosis in PWH are scarce. This study aims to describe the clinical and demographic characteristics along with final diagnosis of hospitalized PWH who had clinical suspicion of progressive disseminated histoplasmosis.

**Methodology/Principal Findings:**

We conducted a multicenter, prospective, cross-sectional study involving 415 hospitalized PWH and clinical suspicion of PDH across ten tertiary care hospitals in Mexico. Participants underwent comprehensive evaluation for *Histoplasmosis* infection, including cultures, histopathology, and urine antigen testing. Of the total cohort, 108 patients (26%) had proven PDH, 162 (39%) received an alternative diagnosis, and 145 (35%) had no definitive diagnosis. In univariate analyses, proven PDH was more frequently associated with skin lesions, thrombocytopenia, elevated AST and LDH levels (>2x ULN), and micronodular infiltrates on chest imaging. In contrast, lymphadenopathy, tree-in-bud patterns, pleural effusion, hepatomegaly, and splenomegaly in imaging were more commonly observed in patients without proven PDH.

Among patients without proven PDH, 41 met the criteria for probable PDH. Compared with proven PDH, probable cases exhibited higher rates of lymphadenopathy (73% vs 50%). Conversely, elevated AST (61% vs 39%) and LDH (74% vs 35%) levels were more frequent in proven PDH cases. While radiographic lung involvement was common in both groups, mediastinal lymphadenopathy (29% vs 12%), pleural effusion (17% vs 3.7%), and hepatomegaly (56% vs 37%) were significantly more frequent in probable PDH cases. Clinical response to antifungal therapy was higher in proven PDH (38% vs 24%), although this difference was not statistically significant.

Compared to patients with tuberculosis (TB) alone, those with proven PDH alone showed more profound immunosuppression, with a greater proportion presenting CD4 + counts <50 cells/mm^3^. Skin lesions, LDH elevation, and micronodular pulmonary infiltrates were also more frequent in the proven PDH group, underscoring their diagnostic relevance. In contrast, lymphadenopathy, tree-in-bud opacities, hepatomegaly, and splenomegaly were more common in TB. Importantly, TB coinfection was present in 13 patients with proven PDH (12%) and in 12 with probable PDH (29%).

In an exploratory analysis of predictors for proven PDH, elevated LDH level was the strongest predictor (adjusted prevalence odds ratio [aPOR] of 6.82, 95% CI 3.56 – 13.4, p < 0.001), followed by the presence of micronodular infiltrates on chest imaging (aPOR 1.94, 95% CI 1.06 – 3.62, p = 0.33). In contrast, pleural effusion on imaging was the strongest negative predictor for proven PDH (aPOR 0.28, 95% CI 0.07 – 0.92, p = 0.0498).

**Conclusions/Significance:**

Histoplasmosis represents a substantial diagnostic burden among PWH in Mexico, particularly in those with advanced disease. Our findings highlight the urgent need to expand access to rapid and sensitive diagnostic tools, improve clinical awareness, and promote routine screening for PDH in PWH presenting with febrile illness, especially in TB-endemic regions. Elevated LDH levels, skin lesions, and micronodular infiltrates on chest imaging were the most useful features to differentiate proven histoplasmosis from tuberculosis and probable histoplasmosis.

## Author summary

Histoplasmosis is a potentially fatal fungal infection in persons with HIV, especially those with advanced disease and low CD4 cell counts. In regions like Latin America, where tuberculosis (TB) is also widespread, histoplasmosis is often misdiagnosed as TB because both diseases share similar symptoms, such as fever, weight loss, and enlarged lymph nodes. Delayed or incorrect diagnosis can prove deadly.

To explore this problem, we studied 415 PWH hospitalized with suspected histoplasmosis at ten Mexican hospitals. We confirmed histoplasmosis in 26% of patients using culture or tissue examination. Key clues—such as skin lesion, elevated lactate dehydrogenase (LDH) and distinctive chest X-ray patterns—helped distinguish histoplasmosis from TB. We also compared “proven” cases (confirmed by culture or histopathology) with “probable” cases (positive urine antigen test) and identified additional clinical features that could guide diagnosis when sophisticated testing is unavailable. Despite extensive work-up, many patients remained undiagnosed, and some received antifungal treatment based on clinical judgment alone.

Our results highlight the urgent need to expand access to accurate diagnostics and antifungal drugs in Mexico and similar settings. Considering histoplasmosis routinely in febrile HIV patients by training clinicians could save lives from this neglected tropical disease.

## Introduction

Histoplasmosis is a systemic fungal infection caused by *Histoplasma* spp., a dimorphic fungus that exists as mold in the environment and transforms into an intracellular yeast form upon infecting humans [1]. Although traditionally considered an endemic mycosis limited to specific geographical areas, growing evidence suggests a broader distribution [2,3]. Global migration, climate change, improved access to diagnostic tools, increased clinical awareness, and the expanding population of immunosuppressed individuals—especially persons with HIV (PWH)—have all contributed to the recognition of histoplasmosis in previously non-endemic regions and to its rising incidence [4,5].

Among immunocompromised individuals, histoplasmosis stands out as a major opportunistic infection. In PWH, the predominant clinical form is progressive disseminated histoplasmosis (PDH), a potentially fatal condition if not promptly diagnosed and treated [6]. The symptoms of PDH are non-specific and frequently mimic those of other infections, particularly disseminated tuberculosis (TB), leading to frequent misdiagnosis [7]. This clinical overlap presents a critical challenge, as PDH requires different management strategies and delays in appropriate treatment can result in poor outcomes [8].

Throughout Latin America and the Caribbean, histoplasmosis ranks among the most common fungal opportunistic infections in persons with HIV [9]. A systematic review found that 14% of symptomatic PWH have detectable *Histoplasma* antigenuria [10]. This mycosis accounts for an estimated 5%-15% of all advanced HIV disease-related deaths each year in the region [7]. Indeed, most of the available data originate from Latin America and the Caribbean—where approximately 15,600 new cases and 4,500 deaths are recorded each year among PWH—and suggest that histoplasmosis incidence in the region is comparable to that of tuberculosis in PWH, with potentially even higher mortality [9]. Supporting this concern, a large multicenter cohort study conducted in the region among patients with clinical suspicion of TB found a 60% increase in culture-negative compared to culture-positive TB cases. Furthermore, patients with culture-negative TB had a 79% higher risk of death [11]. These findings suggest that a substantial proportion of these culture-negative cases may have been undiagnosed histoplasmosis, mistakenly treated as TB [12]. In Mexico, epidemiological evidence on histoplasmosis is scarce and remains in need of updating, particularly regarding persons with HIV [13–15].

Despite global progress in antiretroviral therapy (ART) coverage, a considerable number of PWH continue to present with advanced HIV disease (AHD)—defined by a CD4 cell count <200 cells/mm³ [16]. In 2023, UNAIDS/WHO estimated that 39.9 million people were living with HIV worldwide [17]. In Latin America and the Caribbean, over 30% of newly diagnosed patients enter care with AHD [7], highlighting persistent gaps in early detection. PDH imposes a heavy toll in this population: regional cohorts report mortality rates of up to 30% [18], and in a large Guatemalan study, *Histoplasma* counted for 31% of all opportunistic infections and was the most frequent defining manifestation of advanced HIV [19]. However, it remains underdiagnosed, largely due to its clinical resemblance to TB and the limited availability of sensitive diagnostic tools. This diagnostic gap likely contributes to the high mortality associated with PDH, especially among those with AHD. Accurate epidemiological data are still lacking in many settings.

This study aims to describe the clinical and demographic characteristics along with final diagnosis of hospitalized PWH who had clinical suspicion of progressive disseminated histoplasmosis. As a secondary objective, we compare individuals with proven versus probable PDH, as well as those with proven PDH alone versus those with *Mycobacterium tuberculosis* infection alone, to identify features that may aid in the earlier and more accurate recognition of histoplasmosis in the context of advanced HIV disease.

## Materials and methods

### Ethics statement

The study was approved by the Human Ethics and Biomedical Research Committees of the coordinating center (REF: 1626), the National Institute of Medical Sciences and Nutrition Salvador Zubiran, as well as by the institutional review boards of each participating center. The study was conducted in accordance with the principles expressed in the Declaration of Helsinki. Written informed consent was obtained from all subjects involved in the study.

### Study design

We conducted a cross-sectional study using clinical and demographic data from participants enrolled in a parent study designed to evaluate a novel diagnostic test for progressive disseminated histoplasmosis in PWH [20,21]. Participants were prospectively recruited in ten centers located in central west, central, and southeast regions of the country.

### Participants

The study population consisted of adult patients (≥18 years) with confirmed HIV infection who were hospitalized at one of ten tertiary hospitals in Mexico and met criteria for suspected progressive disseminated histoplasmosis. Suspicion of PDH was defined as the presence of at least three of the following clinical, laboratory, or radiographic findings: fever, weight loss (>5% over six months), lymphadenopathy, hepatomegaly, splenomegaly, mucosal ulcers, skin lesions, gastrointestinal bleeding, diarrhea; bicytopenia or pancytopenia (hemoglobin <10 g/dL, neutrophils <1.8 x10^3^/μL, platelets <100,000/μL); elevated levels (≥2x upper limit of normal) of aspartate aminotransferase, lactic dehydrogenase, or ferritin; or imaging findings consistent with extrapulmonary organ involvement (e.g., lymphadenopathy, hepatosplenomegaly, abscesses, or focal lesions in the pancreas, gastrointestinal tract, adrenal glands, central nervous system, or other organs) [20,21].

### Diagnostic workup

Upon enrollment, all patients were evaluated by infectious disease specialists, who determined the diagnostic workup and treatment based on clinical judgment and local protocols. Specialists completed standardized case report forms documenting demographic data, risk factors (e.g., occupational exposures, travel history, zoonotic contact, or laboratory work), HIV-related variables (e.g., time since diagnosis, most recent CD4 count and viral load, current ART regimen), comorbidity burden (Charlson Index), history of immunosuppressive therapy, concurrent infections or malignancies, signs and symptoms at presentation, initial laboratory and imaging findings, and if the patient received empiric antifungal therapy within 72 hours of admission with any of fluconazole, itraconazole, voriconazole, amphotericin B, or echinocandins.

When clinically indicated, biological samples—including peripheral blood, bone marrow aspirates, and tissue biopsies—were collected for culture and histopathological analysis. All available specimens, including histopathological samples, were sent to, and processed at the central reference laboratory and Pathology Department of the National Institute of Medical Sciences and Nutrition Salvador Zubiran in Mexico City. Urine antigen detection was performed using three commercial assays: the IMMY ALPHA *Histoplasma* EIA (IMMY, Norman, OK), the IMMY *Histoplasma* Galactomannan ASR EIA, and the Miravista *Histoplasma* Lateral Flow Assay. All procedures were conducted according to the manufacturers’ instructions and previously published protocols [20,21]. Imaging studies were performed and interpreted locally by radiologists at each participating hospital.

### Definitions

Proven PDH was defined according to the EORTC/MSGREC revised criteria as the isolation of *Histoplasma* spp. from culture of a clinical specimen (e.g., blood, bone marrow, or tissue biopsy) or histopathological demonstration of characteristic intracellular yeasts in macrophages. Probable PDH was defined as a case with clinical suspicion of PDH and a positive *Histoplasma* urine antigen (HUAg) test, in the absence of microbiological or histopathological confirmation [22]. Because these cases may include false positives, we did not include them in our primary analysis, which focused on patients with versus without proven PDH. Instead, probable PDH cases were examined separately in secondary analyses to explore how they differ from confirmed cases. Patients were classified as PDH-negative if they had negative fungal cultures, absence of yeasts on histopathology, and negative HUAg results, regardless of other opportunistic infections or AIDS-defining conditions present.

Other etiologies were defined based on workup made by each hospital which included cultures (blood, bone marrow, tissue biopsies), other microbiological methods, and histopathology analysis.

Clinical response was defined as resolution of fever and improvement of initial signs and symptoms within two weeks of empiric antifungal treatment initiation, at the discretion of each infectious disease specialist based on their clinical judgment. Importantly, we did not collect information regarding other antimicrobial treatments received by the study participants.

### Statistical analysis

Baseline characteristics were summarized using frequencies and percentages for categorical variables, and medians with interquartile ranges (IQRs) for continuous variables. These were presented overall and stratified by diagnosis of proven progressive disseminated histoplasmosis. Group comparisons were conducted using the chi-squared test for independence or the Welch’s t-test (allowing for unequal variances), as appropriate.

To address the secondary objective, we compared baseline characteristics between patients with proven versus probable PDH, and between those with proven PDH alone and those with *Mycobacterium tuberculosis* infection alone.

As an exploratory analysis, we aimed to identify factors associated with proven PDH. We selected baseline characteristics that were both clinically relevant and significantly associated with proven PDH in the initial comparisons (unadjusted analysis). For each characteristic, we estimated its prevalence odds ratio (POR) using a univariate logistic regression, with proven PDH as the binary outcome. Subsequently, we estimated adjusted PORs using a multivariable logistic regression, including all selected predictors (adjusted analysis). The multivariable model assumed a linear relationship between the log odds of proven PDH and the predictors. A two-sided p-value < 0.05 was considered statistically significant. All analyses were performed using R version 4.4.3.

## Results

**Fig 1:**
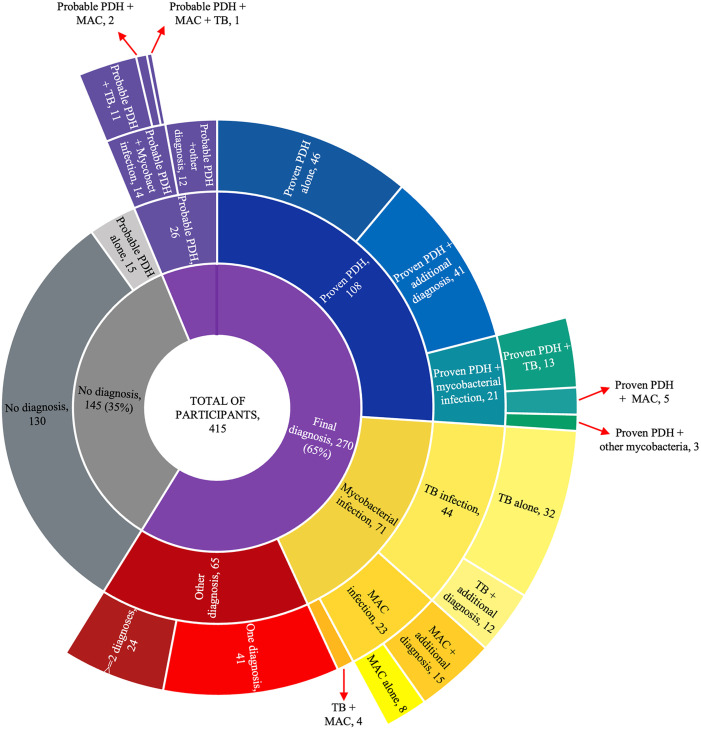
Distribution of the study population by final diagnosis.

Of the 415 participants evaluated, 108 (26%) were diagnosed with proven progressive disseminated histoplasmosis, 162 (39%) were assigned an alternative etiological diagnosis, and in 145 cases (35%) no definitive diagnosis could be made despite an extensive diagnostic workup. Among the 108 proven PDH cases, 21 had mycobacterial coinfection and 41 had an additional non-mycobacterial diagnosis; the remaining 46 had histoplasmosis only. Overall, 73 participants (17.6% of the cohort) were diagnosed with tuberculosis: 13 were coinfected with proven PDH, 12 with probable histoplasmosis, 32 with tuberculosis alone, 12 with tuberculosis plus another non-PDH diagnosis, and 4 were coinfected with tuberculosis and *Mycobacterium avium complex* (MAC). A detailed description of the final diagnosis is shown in Fig 1.

**Fig 2:**
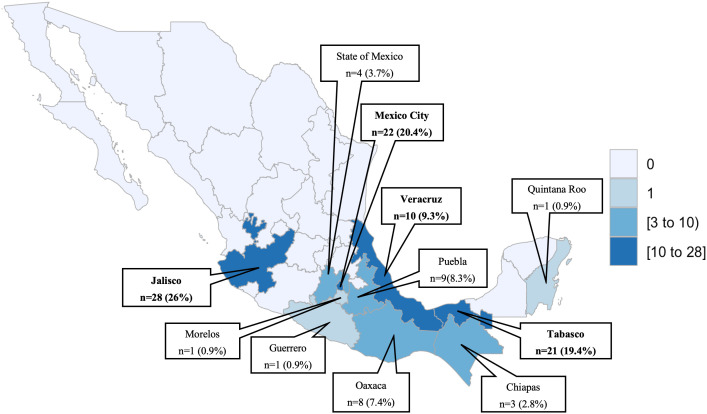
Geographic distribution of proven PDH cases according to place of residency.

The place of residency of all patients with proven PDH is displayed in Fig 2. The states with most proven PDH cases were Jalisco (central-west) with 28 cases (26%), Mexico City (central Mexico) with 22 cases (20%), Tabasco (south-east) with 21 cases (19%), and Veracruz (central-east) with 10 cases (9%).

### Clinical and demographic differences in patients with and without proven PDH

The comparison between patients with proven PDH versus those without proven PDH is provided in Table 1. Patients with proven PDH were slightly younger than those without (median age 33 vs. 36 years; *p*=0.030). Although median CD4+ T-cell counts were statistically significantly different (28 vs. 38 cells/mm³; *p*=0.027), such difference was not clinically relevant. Moreover, both groups exhibited advanced immunosuppression, with a high proportion of participants in each group presenting with CD4+ counts <50 cells/mm³ (69% vs. 58%; *p*=0.050).

**Table 1:** Baseline characteristics of study participants, overall and stratified by diagnosis of proven progressive disseminated histoplasmosis.

Characteristic	N	Overall, N = 415	Proven progressive disseminated histoplasmosis, N = 108	No histoplasmosis, N = 307	P-value
Age (years)	414	35 (28, 42)	33 (27, 38)	36 (29, 43)	**0.030**
Male	415	360 (87%)	90 (83%)	270 (88%)	0.2
**HIV infection**					
Time with diagnosis (months)	405	2 (0, 35)	2 (0, 27)	2 (0, 42)	0.2
ART-naive	414	227 (55%)	62 (57%)	165 (54%)	0.5
CD4+ (cells/mm^3^)	376	34 (13, 81)	28 (11, 64)	38 (14, 86)	**0.027**
CD4 + count < 50 cells/mm^3^	376	227 (60%)	61 (69%)	166 (58%)	**0.050**
Log HIV viral load	371	12.2 (10.2, 13.2)	11.9 (10.5, 13.0)	12.3 (10.0, 13.5)	0.8
**Symptoms/signs**					
Fever	414	394 (95%)	107 (99%)	287 (94%)	0.028
Weight loss	415	372 (90%)	98 (91%)	274 (89%)	0.7
Hepatomegaly	415	291 (70%)	71 (66%)	220 (72%)	0.2
Lymphadenopathy	415	261 (63%)	54 (50%)	207 (67%)	**0.001**
Splenomegaly	415	224 (54%)	54 (50%)	170 (55%)	0.3
Cough	415	254 (61%)	65 (60%)	189 (62%)	0.8
Dyspnea	415	219 (53%)	59 (55%)	160 (52%)	0.7
Diarrhea	415	204 (49%)	55 (51%)	149 (49%)	0.7
Skin lesions	415	98 (24%)	35 (32%)	63 (21%)	**0.012**
Vomiting	415	87 (21%)	30 (28%)	57 (19%)	**0.043**
Acute kidney injury	415	70 (17%)	23 (21%)	47 (15%)	0.2
Mucosal ulcers	415	52 (13%)	12 (11%)	40 (13%)	0.6
Gastrointestinal bleeding	415	50 (12%)	11 (10%)	39 (13%)	0.5
**Laboratory abnormalities**					
Bicytopenia or pancytopenia	407	170 (42%)	53 (50%)	117 (39%)	0.058
Platelet count < 100 x 10^3^/µL	407	160 (39%)	58 (54%)	102 (34%)	**<0.001**
AST > 2xULN	387	145 (37%)	63 (61%)	82 (29%)	**<0.001**
LDH > 2xULN	342	127 (37%)	71 (74%)	56 (23%)	**<0.001**
**Radiographic findings**					
Evidence of lung involvement	414	289 (70%)	73 (68%)	216 (71%)	0.6
Micronodular infiltrate	413	193 (47%)	60 (56%)	133 (43%)	**0.024**
Mediastinal lymphadenopathy	413	92 (22%)	13 (12%)	79 (26%)	**0.003**
Tree-in-bud infiltrate	413	68 (16%)	11 (10%)	57 (19%)	**0.045**
Ground-glass opacity	413	30 (7.3%)	5 (4.7%)	25 (8.2%)	0.2
Pleural effusion	413	36 (8.7%)	4 (3.7%)	32 (10%)	**0.034**
Evidence of extrapulmonary involvement	413	284 (69%)	52 (49%)	232 (76%)	**<0.001**
Hepatomegaly	413	224 (54%)	40 (37%)	184 (60%)	**<0.001**
Splenomegaly	413	186 (45%)	34 (32%)	152 (50%)	**0.001**
**Antifungal therapy**					
Empiric antifungal therapy within 72 hours of admission^¶^	415	150 (36%)	53 (49%)	97 (32%)	**0.001**
Clinical response within two weeks^⋄^	137	31 (23%)	18 (38%)	13 (15%)	**0.002**

Summaries reported in median (interquartile range) or n (%). ART, Antiretroviral therapy; ULN, upper limit of normal.

^¶^Included any of fluconazole, itraconazole, voriconazole, amphotericin B, and echinocandins.

^⋄^Clinical response was defined as resolution of fever and improvement of initial signs and symptoms within two weeks. Only assessed in those that received empiric antifungal therapy within 72 hours of admission.

Fever was reported in nearly all participants, consistent with its inclusion as a key eligibility criterion, without a clinically relevant difference between patients with versus without proven PDH (99% vs. 94%; *p*=0.028). Lymphadenopathy was more frequent among those without proven PDH (67% vs. 50%; *p*=0.001), while skin lesions (32% vs. 21%; *p*=0.012) and vomiting (28% vs. 19%; *p*=0.043) were more common in proven PDH cases. No statistically significant differences were found in the frequency of diarrhea, dyspnea, or mucosal ulcers.

Laboratory abnormalities were more pronounced among patients with proven PDH. Thrombocytopenia (platelet count <100 × 10³/μL) was observed in 54% of patients with proven PDH compared to 34% without proven PDH (*p*<0.001). Elevated AST and LDH levels—above two times the upper limit of normal—were significantly more prevalent in the group with proven PDH (AST>2x UNL: 61% vs. 29%; LDH>2x UNL: 74% vs. 23%; both *p*<0.001).

Radiological findings revealed distinct patterns between groups. Micronodular infiltrates were more frequent among proven PDH patients (56% vs. 43%; *p*=0.024), whereas mediastinal lymphadenopathy (12% vs. 26%; *p*=0.003), tree-in-bud patterns (10% vs. 19%; *p*=0.045), and pleural effusions (3.7% vs. 10%; *p*=0.034) were more common in patients without proven PDH. Notably, both clinical and radiographic findings of hepatomegaly and splenomegaly were more frequent in the group without proven PDH (hepatomegaly: 60% vs. 37%, *p*<0.001; splenomegaly: 50% vs. 32%, *p*=0.001).

Empiric antifungal therapy was more frequently initiated in patients with proven PDH (49% vs. 32%; *p*=0.001), and clinical improvement was observed more often in this group (38% vs. 15%; *p*<0.002). Additional information regarding high-risk exposure factors and laboratory measures are provided in S1 Table (Supplement), and the diagnostic modalities for all 108 proven PDH cases—per EORTC/MSG criteria—are illustrated in S1 Fig (Supplement).

### Comparison between proven and probable progressive disseminated histoplasmosis

Among patients without proven PDH, 41 fulfilled criteria for probable PDH. Of these, 14 had coinfection with mycobacteria, 12 had an additional diagnosis different from mycobacterial infection, and 15 had no additional diagnosis (see Fig 1). These 15 cases of probable PDH without an alternative diagnosis were counted in the “no definite diagnosis” group because they originated from a diagnostic accuracy study evaluating urine antigen testing for *Histoplasma* against a gold standard of culture and histopathology. In that study, no comprehensive assessment for other potential etiologies was conducted. Consequently, these cases were classified as false positives, based on negative reference test results and the absence of any confirmed alternative diagnosis.

When comparing signs and symptoms at the initial examination between patients with proven versus probable PDH, only the presence of lymphadenopathy was significantly different, being more common in those with probable PDH (73% vs. 50%; *p*=0.011).

In contrast, regarding laboratory findings, patients with proven PDH showed higher rates of AST and LDH elevation, consistent with greater systemic inflammation or tissue involvement (AST > 2 × ULN: 61% vs. 39%; *p* = 0.025; LDH > 2 × ULN: 74% vs. 35%; *p* < 0.001).

Radiographic lung involvement was frequent in both groups (68% vs. 83%; p = 0.063), but mediastinal lymphadenopathy and pleural effusion were significantly more frequent in those with probable PDH (29% vs. 12%, p = 0.013; and 17% vs. 3.7%, p = 0.011, respectively). In addition, hepatomegaly was more commonly observed in the probable PDH group (56% vs. 37%, p = 0.039).

Use of empirical antifungal therapy was comparable across groups (49% vs. 44%; p = 0.6). Favorable clinical response was more frequently reported in the proven PDH group (38% vs. 24%), although it did not reach statistical significance (p = 0.3). Furthermore, there were less infections with *Mycobacterium tuberculosis* in the proven PDH group (12% vs. 29%, p = 0.012). The detailed comparison between patients with proven versus probable PDH is provided in Table 2.

**Table 2:** Comparison of baseline characteristics of participants with proven versus probable histoplasmosis.

Characteristic	N	Proven progressive disseminated histoplasmosis, N = 108	Probable progressive disseminated histoplasmosis, N = 41	P-value
Age (years)	149	33 (27, 38)	36 (29, 43)	0.12
Male	149	90 (83%)	37 (90%)	0.3
**HIV infection**				
Time with diagnosis (months)	146	2 (0, 27)	2 (0, 8)	>0.9
ART-naive	149	62 (57%)	23 (56%)	0.9
CD4+ (cells/mm^3^)	126	28 (11, 64)	41 (13, 79)	0.12
CD4 + count < 50 cells/mm^3^	126	61 (69%)	22 (58%)	0.2
Log HIV viral load	124	11.9 (10.5, 13.0)	12.8 (9.0, 14.1)	0.8
**Symptoms/signs**				
Fever	149	107 (99%)	41 (100%)	>0.9
Weight loss	149	98 (91%)	39 (95%)	0.5
Hepatomegaly	149	71 (66%)	27 (66%)	>0.9
Lymphadenopathy	149	54 (50%)	30 (73%)	**0.011**
Splenomegaly	149	54 (50%)	15 (37%)	0.14
Cough	149	65 (60%)	31 (76%)	0.079
Dyspnea	149	59 (55%)	25 (61%)	0.5
Diarrhea	149	55 (51%)	24 (59%)	0.4
Skin lesions	149	35 (32%)	8 (20%)	0.12
Vomiting	149	30 (28%)	9 (22%)	0.5
Acute kidney injury	149	23 (21%)	7 (17%)	0.6
Mucosal ulcers	149	12 (11%)	4 (9.8%)	>0.9
Gastrointestinal bleeding	149	11 (10%)	4 (9.8%)	>0.9
**Laboratory abnormalities**				
Bicytopenia or pancytopenia	148	53 (50%)	23 (56%)	0.5
Platelet count < 100 x 10^3^/µL	148	58 (54%)	20 (49%)	0.6
AST > 2xULN	142	63 (61%)	15 (39%)	**0.025**
LDH > 2xULN	130	71 (74%)	12 (35%)	**<0.001**
**Radiographic findings**				
Evidence of lung involvement	149	73 (68%)	34 (83%)	0.063
Micronodular infiltrate	148	60 (56%)	21 (51%)	0.6
Mediastinal lymphadenopathy	148	13 (12%)	12 (29%)	**0.013**
Tree-in-bud infiltrate	148	11 (10%)	5 (12%)	0.8
Ground-glass opacity	148	5 (4.7%)	0 (0%)	0.3
Pleural effusion	148	4 (3.7%)	7 (17%)	**0.011**
Evidence of extrapulmonary involvement	148	52 (49%)	29 (71%)	**0.015**
Hepatomegaly	148	40 (37%)	23 (56%)	**0.039**
Splenomegaly	148	34 (32%)	14 (34%)	0.8
**Antifungal therapy**				
Empiric antifungal therapy within 72 hours of admission^¶^	149	53 (49%)	18 (44%)	0.6
Clinical response within two weeks^⋄^	65	18 (38%)	4 (24%)	0.3
**Mycobacterium tuberculosis infection**	149	13 (12%)	12 (29%)	0.012

Summaries reported in median (interquartile range) or n (%). ART, Antiretroviral therapy; ULN, upper limit of normal.

^¶^Included any of fluconazole, itraconazole, voriconazole, amphotericin B, and echinocandins.

^⋄^Clinical response was defined as resolution of fever and improvement of initial signs and symptoms within two weeks. Only assessed in those that received empiric antifungal therapy within 72 hours of admission.

### Comparison between patients with proven PDH alone and those with *Mycobacterium tuberculosis* infection alone

There were 46 patients with proven PDH alone and 32 with *Mycobacterium tuberculosis* infection alone, besides HIV diagnosis. Compared to patients with tuberculosis, those with proven PDH alone had a significantly higher proportion of severe immunosuppression, with 74% presenting CD4 + counts <50 cells/mm³ versus 48% in the TB alone group (*p* = 0.033). Skin lesions were notably more frequent in those with proven PDH alone (37% vs. 16%; *p* = 0.039), while lymphadenopathy was significantly more common in those with TB alone (88% vs. 50%; *p* < 0.001).

Biochemically, LDH elevation >2 × ULN occurred much more often in proven PDH alone patients (65% vs. 27%; *p* = 0.002), reinforcing its potential diagnostic value. In contrast, radiologically, tree-in-bud infiltrates, hepatomegaly, and splenomegaly were all more frequently in the TB alone group (28% vs. 6.7%, *p* = 0.022; 53% vs. 30%, *p* = 0.044; and 53% vs. 28%, *p* = 0.026; respectively). Table 3 presents a detailed comparison between patients diagnosed solely with proven PDH and those with tuberculosis infection alone.

**Table 3:** Comparison of baseline characteristics between patients diagnosed with only proven PDH vs those with only *Mycobacterium tuberculosis* infection besides HIV diagnosis.

Characteristic	N	Proven progressive disseminated histoplasmosis, N = 46	*M. tuberculosis* infection, N = 32	P-value
Age (years)	78	32 (27, 35)	35 (28, 43)	0.14
Male	78	40 (87%)	28 (88%)	>0.9
**HIV infection**				
Time with diagnosis (months)	75	2 (0, 25)	3 (0, 47)	0.4
ART-naive	78	29 (63%)	18 (56%)	0.5
CD4+ (cells/mm^3^)	67	23 (9, 62)	50 (22, 105)	0.3
CD4 + count < 50 cells/mm^3^	67	28 (74%)	14 (48%)	**0.033**
Log HIV viral load	65	11.9 (10.8, 12.9)	12.0 (10.6, 13.3)	0.4
**Symptoms/signs**				
Fever	78	46 (100%)	30 (94%)	0.2
Weight loss	78	39 (85%)	26 (81%)	0.7
Hepatomegaly	78	33 (72%)	24 (75%)	0.7
Lymphadenopathy	78	23 (50%)	28 (88%)	**<0.001**
Splenomegaly	78	24 (52%)	19 (59%)	0.5
Cough	78	28 (61%)	21 (66%)	0.7
Dyspnea	78	23 (50%)	19 (59%)	0.4
Diarrhea	78	26 (57%)	16 (50%)	0.6
Skin lesions	78	17 (37%)	5 (16%)	**0.039**
Vomiting	78	14 (30%)	9 (28%)	0.8
Acute kidney injury	78	7 (15%)	8 (25%)	0.3
Mucosal ulcers	78	4 (8.7%)	3 (9.4%)	>0.9
Gastrointestinal bleeding	78	5 (11%)	4 (13%)	>0.9
**Laboratory abnormalities**				
Bicytopenia or pancytopenia	78	21 (46%)	12 (38%)	0.5
Platelet count < 100 x 10^3^/µL	78	22 (48%)	11 (34%)	0.2
AST > 2xULN	73	25 (57%)	13 (45%)	0.3
LDH > 2xULN	69	28 (65%)	7 (27%)	**0.002**
**Radiographic findings**				
Evidence of lung involvement	78	29 (63%)	24 (75%)	0.3
Micronodular infiltrate	77	27 (60%)	14 (44%)	0.2
Mediastinal lymphadenopathy	77	5 (11%)	9 (28%)	0.056
Tree-in-bud infiltrate	77	3 (6.7%)	9 (28%)	**0.022**
Ground-glass opacity	77	0 (0%)	2 (6.3%)	0.2
Pleural effusion	77	1 (2.2%)	3 (9.4%)	0.3
Evidence of extrapulmonary involvement	78	19 (41%)	25 (78%)	**0.001**
Hepatomegaly	78	14 (30%)	17 (53%)	**0.044**
Splenomegaly	78	13 (28%)	17 (53%)	**0.026**
**Antifungal therapy**				
Empiric antifungal therapy within 72 hours of admission^¶^	78	19 (41%)	8 (25%)	0.14
Clinical response within 2 weeks^⋄^	23	6 (40%)	3 (38%)	>0.9

Summaries reported in median (interquartile range) or n (%). ART, Antiretroviral therapy; ULN, upper limit of normal.

^¶^Included any of fluconazole, itraconazole, voriconazole, amphotericin B, and echinocandins.

^⋄^Clinical response was defined as resolution of fever and improvement of initial signs and symptoms within two weeks. Only assessed in those that received empiric antifungal therapy within 72 hours of admission.

### Factors associated with proven progressive disseminated histoplasmosis

In the analysis of predictors for proven PDH (Table 4), the strongest association was observed for LDH levels >2 × ULN, which remained highly significant after adjustment (aPOR of 6.82; 95% CI: 3.56–13.4; p < 0.001). The presence of micronodular pulmonary infiltrates on imaging was also associated with proven PDH (aPOR of 1.94; 95% CI: 1.06–3.62; p = 0.033). In contrast, pleural effusion was associated with a lower likelihood of proven PDH (aPOR of 0.28; 95% CI: 0.07–0.92; p = 0.0498).

**Table 4:** Exploratory analysis of factors associated with progressive disseminated histoplasmosis.

Characteristic	Unadjusted analysis			Adjusted analysis		
	POR	95% CI	P-value	aPOR	95% CI	P-value
**Signs**						
Lymphadenopathy	0.48	0.31 – 0.75	0.001	0.66	0.35 – 1.22	0.18
Skin lesions	1.86	1.13 – 3.02	0.013	1.46	0.74 – 2.83	0.27
**Laboratory abnormalities**						
Platelet count < 100 x 10^3^/µL	2.30	1.47 – 3.61	< 0.001	1.60	0.88 – 2.89	0.12
AST > 2xULN	3.77	2.37 – 6.06	< 0.001	1.68	0.86 – 3.23	0.12
LDH > 2xULN	9.64	5.66 – 16.9	< 0.001	6.82	3.56 – 13.4	**< 0.001**
**Radiographic findings**						
Micronodular infiltrate	1.66	1.07 – 2.60	0.025	1.94	1.06 – 3.62	**0.033**
Tree-in-bud infiltrate	0.50	0.24 – 0.96	0.048	0.50	0.20 – 1.18	0.12
Pleural effusion	0.33	0.10 – 0.86	0.043	0.28	0.07 – 0.92	**0.0498**
Hepatomegaly	0.40	0.25 – 0.62	< 0.001	0.52	0.21 – 1.24	0.15
Splenomegaly	0.47	0.29 – 0.74	0.002	0.84	0.36 – 2.02	0.69

CI, confidence interval; POR, prevalence odds ratio; aPOR, adjusted prevalence odds ratio; ULN, upper limit of normal.

Other variables—including lymphadenopathy, skin lesions, thrombocytopenia, elevated AST, tree-in-bud infiltrate, hepatomegaly, and splenomegaly—were positively or negatively associated with proven PDH in unadjusted models but did not retain statistical significance after adjustment for other associated predictors.

## Discussion

To our knowledge, this multicenter prospective study is the largest evaluation to date of hospitalized adults with HIV and clinical suspicion of progressive disseminated histoplasmosis in Latin America. Among 415 participants, we confirmed 108 cases of proven PDH, corresponding to a prevalence of 26%. This figure is noteworthy because it exceeds the rates reported in comparable regional cohorts and is nearly double the 13.7% prevalence described in Brazil [23]. Such findings underscored the need for heightened clinical vigilance and strengthened diagnostic capacity in settings that care for patients with advanced HIV disease. Importantly, proven PDH emerged as the leading etiological diagnosis among febrile participants, accounting for 38% of all cases with a defined cause, followed by *Mycobacterium tuberculosis* and *Pneumocystis jirovecii* pneumonia.

Our comparison of proven versus probable PDH cases provides valuable insights into interpreting positive urine antigen tests for *Histoplasma* spp. in the absence of culture or histopathology confirmation. Among the 41 probable PDH cases, 15 had no alternative diagnosis and were categorized under “no definite diagnosis”, while 26 had a confirmed pathogen or comorbidity and were grouped under “final diagnosis”. Clinically, proven PDH cases exhibited more pronounced systemic inflammation, with higher AST and LDH levels. In contrast, probable cases presented with lymphadenopathy, pleural effusion, and hepatomegaly more frequently. Notably, *Mycobacterium* infection was significantly more common among probable PDH cases compared to proven PDH cases (29% vs. 12%, p = 0.012). Overall, the clinical profile of probable PDH cases more closely resemble that of tuberculosis than proven PDH, suggesting that other underlying etiologies might have been missed due to the diagnostic workup focused on histoplasmosis. These clinical differences may offer useful guidance for interpreting positive urine antigen results, particularly while awaiting confirmatory testing or in settings where culture and histopathology—the diagnostic gold standards—are unavailable.

Our findings on *Mycobacterium tuberculosis* infection among patients with proven or probable PDH are consistent with previous reports from Latin America. If we pool proven and probable PDH cases together, 16.7% (25 cases) of our cohort presented with co-infection of TB and PDH (see S2 Table for details) (Supplement). In a prospective cohort study in Brazil that included 570 hospitalized adults with HIV, fever, and additional clinical findings, co-infection with tuberculosis and PDH was reported in 19 cases, representing 15.4% of their proven/probable PDH cases [23]. Similar rates of co-infection have been reported in other countries in the region, including Guatemala [24] (13.1% among proven and probable cases), French Guiana [25] (8% among proven cases), and Panama [26] (15.4% among proven cases). Notably, most TB diagnoses in our cohort were made at the referring hospitals, typically based on respiratory specimens or tissue biopsies. However, in several cases, *Mycobacterium tuberculosis* was also isolated from blood or bone marrow cultures collected during the diagnostic workup for histoplasmosis. This finding reflects a high degree of immunosuppression and disease dissemination in this population and reinforces the need for comprehensive diagnostic strategies that can detect multiple opportunistic infections.

One of the main challenges in diagnosing PDH is its clinical overlap with tuberculosis, a particularly common issue in high-burden settings [27,28]. This overlap in symptoms, laboratory findings, and radiologic features often leads to misdiagnosis and the empirical initiation of anti-tuberculosis treatment, with histoplasmosis being recognized only postmortem in some cases [29]. To better understand this overlap, we compared 46 patients with isolated proven PDH to 32 with isolated TB. We identified distinct patterns: lymphadenopathy, tree-in-bud infiltrates, hepatosplenomegaly, and pleural effusion were more common in TB; whereas skin lesions, cytopenias, and elevated LDH were more frequently observed in proven PDH cases. These findings underscore the diagnostic complexity and emphasize the need for increased clinical suspicion and expanded access to fungal diagnostics.

In our exploratory analysis to identify clinical predictors for proven PDH, an LDH > 2x ULN emerged as the strongest independent predictor (aPOR 6.82), followed by the presence of micronodular pulmonary infiltrates. Conversely, pleural effusion was inversely associated with proven PDH. While other variables such as thrombocytopenia, AST elevation, and skin lesions were significant in unadjusted analyses, their associations diminished after adjustment, likely due to the limited number of patients presenting with these findings. Further research in how to integrate preliminary findings, such as positive urine antigen tests, with clinical predictors may help clinicians improve diagnostic accuracy and more reliably identify cases of proven PDH.

The study was strengthened by a nationwide network of infectious disease specialists and a standardized diagnostic approach including antigen detection, culture, and histopathological analysis. However, several limitations must be acknowledged. Diagnostic tests and treatment strategies were selected by the attending physicians at each site, introducing variability and potential selection bias. Additionally, we did not collect complete outcome data, such as mortality, as the primary focus was diagnostic accuracy. Finally, although our study included ten hospitals, they were in only seven of Mexico’s 32 states, limiting the generalizability of our findings. Future multicenter studies with harmonized diagnostic protocols and longitudinal follow-up will be essential to validate these findings.

In Mexico, the adult HIV prevalence among individuals aged 15–49 years is estimated at approximately 0.3% [30]. Antiretroviral therapy (ART) is provided free of charge by the federal government [31]. Nonetheless, a significant proportion of people living with HIV continue to present with advanced disease (defined as CD4 cell counts bellow 200 cells/mm^3^) [32]. In our cohort, 93% of participants had CD4 cell counts below this threshold, and 61% had counts below 50 cells/mm³. These figures markedly exceed national estimates, which indicate that 43% of newly diagnosed individuals present with advanced HIV disease, [33]. Despite a median time of two months since HIV diagnosis, more than half of the participants had not initiated ART at the time of enrollment. These findings underscore ongoing challenges in achieving timely HIV diagnosis, linkage to care, and treatment initiation—critical steps in the HIV care cascade that remain key areas for improvement within Mexico´s national HIV response.

Regarding histoplasmosis management, access to optimal diagnostic and therapeutic tools remains limited. *Histoplasma* urine antigen testing—added to the WHO Essential Diagnostics List in 2019—is not yet widely available in most public hospitals [34]. Similarly, liposomal amphotericin B, the treatment of choice for proven PDH, is unavailable in most healthcare centers, and access to BSL-3 laboratory infrastructure required for fungal cultures is scarce. These structural limitations contribute to underdiagnosis and inadequate treatment, perpetuating poor outcomes in patients with advanced HIV disease [35,36].

Our findings support the call to action proposed by the Manaus Declaration [37] and the Porto Alegre Manifesto [36], which advocate for universal access to rapid diagnostic testing and effective treatment for histoplasmosis across the Americas. As a direct result of this study, our institution has established reference capabilities for *Histoplasma* urine antigen testing, enabling sample processing from hospitals nationwide. These efforts are aligned with PAHO guidelines for the diagnosis and management of disseminated histoplasmosis in persons with HIV [7].

## Conclusions

This multicenter study highlights the significant burden and diagnostic complexity of progressive disseminated histoplasmosis among hospitalized adults with HIV in Mexico. Our findings underscore the critical need for heightened clinical suspicion and access to sensitive diagnostic tools to improve recognition of PDH in advanced HIV disease. Distinct clinical and laboratory features, including elevated LDH and specific radiographic findings, may help distinguish PDH from tuberculosis, guiding early management in settings with limited diagnostic capacity. The stratification into proven and probable PDH, based on standardized criteria, further emphasizes the diagnostic utility of antigen testing in real-world practice. Structural barriers such as limited access to fungal cultures, antigen testing, and liposomal amphotericin B continue to hinder timely diagnosis and treatment. Addressing these gaps through expanded diagnostic infrastructure, increased awareness, and integration of fungal disease management into national HIV programs is essential to reduce morbidity and mortality form histoplasmosis in the region.

## Supporting information

S1 Fig Venn diagram and corresponding specimen-type matrix for the 108 patients with proven PDH. (TIFF)

S1 Table Additional baseline characteristics of study participants regarding high-risk exposure factors and laboratory measures. (DOCX)

S2 Table Characteristics of the twenty-five PWH coinfected with histoplasmosis and tuberculosis. (DOCX)
