# Supplementary figures and images for: Differentiating proven progressive disseminated histoplasmosis from other diagnoses in hospitalized persons with HIV and suspected progressive disseminated histoplasmosis: Findings from a clinical and demographic study in Mexico
Source: PLoS Negl Trop Dis. 2025 Sep 17;19(9):e0013527. doi: 10.1371/journal.pntd.0013527 (PMC12453177; doi:10.1371/journal.pntd.0013527)

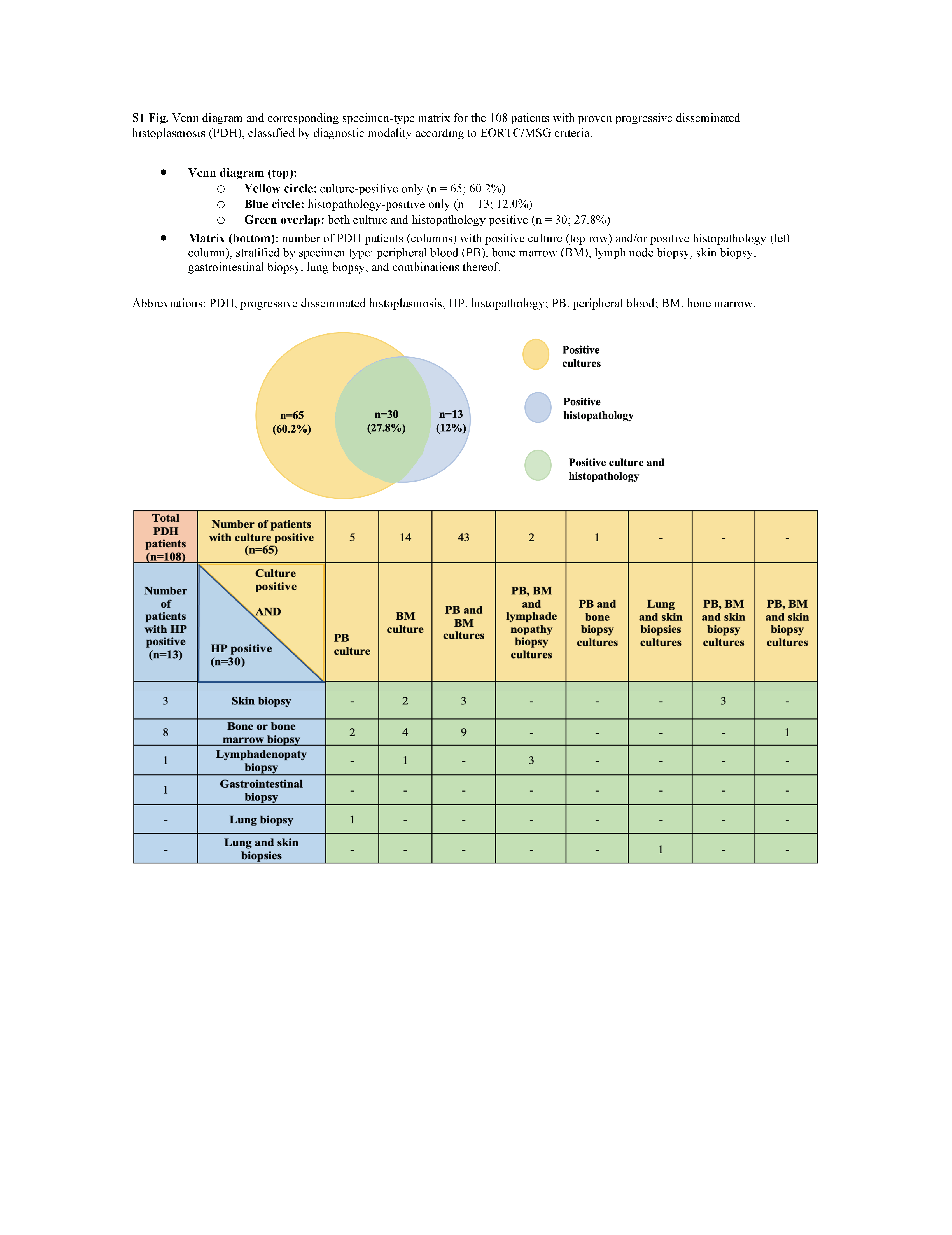

Supplement: S1 Fig — (TIFF) [file pntd.0013527.s001.tiff]
