# Supplementary material for: Differentiating proven progressive disseminated histoplasmosis from other diagnoses in hospitalized persons with HIV and suspected progressive disseminated histoplasmosis: Findings from a clinical and demographic study in Mexico
Source: PLoS Negl Trop Dis. 2025 Sep 17;19(9):e0013527. doi: 10.1371/journal.pntd.0013527 (PMC12453177; doi:10.1371/journal.pntd.0013527)
Supplement: S1 Table — (DOCX) [file pntd.0013527.s002.docx]

S1 Table. Additional baseline characteristics of study participants, overall and stratified by diagnosis of proven progressive disseminated histoplasmosis.

| **Characteristic** | **N** | **Overall, N = 415** | **Proven progressive disseminated histoplasmosis,**  **N = 108** | **No histoplasmosis, N = 307** | **P-value** |
| --- | --- | --- | --- | --- | --- |
| **High-Risk Exposure Factors for Histoplasmosis** |  |  |  |  |  |
| Contact with birds | 414 | 124 (30%) | 31 (29%) | 93 (30%) | 0.7 |
| Contact with bats | 414 | 30 (7.2%) | 9 (8.3%) | 21 (6.9%) | 0.6 |
| Exposure to storage buildings | 414 | 93 (22%) | 14 (13%) | 79 (26%) | 0.006 |
| Occupational exposure | 414 | 54 (13%) | 15 (14%) | 39 (13%) | 0.8 |
| Laboratory-related exposure | 414 | 1 (0.2%) | 0 (0%) | 1 (0.3%) | >0.9 |
| Participation in ecoturism | 414 | 72 (17%) | 21 (19%) | 51 (17%) | 0.5 |
| History of prior exposure | 414 | 14 (3.4%) | 6 (5.6%) | 8 (2.6%) | 0.2 |
| **Comorbidity and Laboratory Measures** |  |  |  |  |  |
| Charlson Comorbidity Index > 3 | 415 | 398 (96%) | 108 (100%) | 290 (94%) | 0.009 |
| Leukocytes count (x10^3^/µL) | 408 | 3.9 (2.4, 6.5) | 3.4 (2.1, 4.7) | 4.1 (2.7, 6.9) | 0.13 |
| Neutrophil count (x10^3^/µL) | 401 | 2.7 (1.6, 4.5) | 2.6 (1.5, 4.0) | 2.8 (1.6, 5.1) | 0.4 |
| Platelet count (100 x10^3^/µL) | 407 | 141 (65, 242) | 90 (51, 186) | 154 (72, 255) | 0.004 |
| Creatinine (mg/dL) | 402 | 0.82 (0.63, 1.19) | 0.82 (0.62, 1.31) | 0.82 (0.63, 1.16) | 0.8 |
| Blood urea nitrogen (mg/dL) | 336 | 15 (10, 25) | 15 (9, 25) | 15 (10, 25) | 0.8 |
| Total bilirubin (mg/dL) | 384 | 0.66 (0.45, 1.14) | 0.70 (0.50, 1.31) | 0.62 (0.41, 1.08) | 0.7 |
| Alanine aminotransferase (U/L) | 386 | 39 (22, 62) | 46 (26, 70) | 37 (21, 59) | >0.9 |
| Aspartate aminotransferase (U/L) | 386 | 39 (22, 62) | 46 (26, 70) | 37 (21, 59) | >0.9 |
| Alkaline phosphatase (U/L) | 369 | 144 (87, 284) | 223 (115, 406) | 127 (84, 235) | 0.002 |
| Lactate dehydrogenase (U/L) | 342 | 394 (217, 813) | 970 (537, 2,188) | 304 (193, 517) | <0.001 |

Summaries reported in median (interquartile range) or n (%).
