# Supplementary material for: Differentiating proven progressive disseminated histoplasmosis from other diagnoses in hospitalized persons with HIV and suspected progressive disseminated histoplasmosis: Findings from a clinical and demographic study in Mexico
Source: PLoS Negl Trop Dis. 2025 Sep 17;19(9):e0013527. doi: 10.1371/journal.pntd.0013527 (PMC12453177; doi:10.1371/journal.pntd.0013527)
Supplement: S2 Table — (DOCX) [file pntd.0013527.s003.docx]

|  | **Sex and age (years)** | **Months of HIV diagnosis** | **Naive to ARV**  **treatment** | **CD4+**  **cell count (cells/μ**  **L)** | **Method of tuberculosis diagnosis** | **Method of proven histoplasmosis** | **Positive Histoplasma urine antigen test** | **Additional diagnosis** |
| --- | --- | --- | --- | --- | --- | --- | --- | --- |
| 1 | Female, 19 | 3 | No | 59 | Hospital of origin | Histopathology (bone marrow) | - |  |
| 2 | Female, 24 | 35 | No | 30 | Hospital of origin | Culture (blood and  lymphadenopathy biopsy) | Immy alpha, immy GM, Miravista |  |
| 3 | Female, 25 | 4 | No | 34 | Hospital of origin | Culture (blood and bone marrow) | Immy alpha, immy GM, Miravista |  |
| 4 | Male, 23 | 1 | No | 9 | Hospital of origin | Culture (blood and bone marrow) | Immy alpha, immy GM, Miravista |  |
| 5 | Male, 24 | 9 | No | NA | Hospital of origin | Culture (blood and bone marrow)  and histopathology (bone marrow) | Immy alpha, immy GM, Miravista |  |
| 6 | Male, 27 | 1 | Yes | 16 | Hospital of origin | Culture (blood and bone marrow) | Immy GM and Miravista |  |
| 7 | Male, 30 | 8 | No | 178 | Hospital of origin | Culture (blood) and histopathology  (skin) | Immy GM and Miravista |  |
| 8 | Male, 33 | 2 | Yes | 45 | Hospital of origin | Culture (blood and bone marrow) | Immy GM and Miravista |  |
| 9 | Male, 37 | 0 | Yes | 22 | Hospital of origin | Culture (blood and bone marrow) | Miravista |  |
| 10 | Male, 38 | 6 | No | 65 | Hospital of origin | Culture (blood and bone marrow) | Immy alpha, immy GM, Miravista |  |
| 11 | Male, 39 | 3 | Yes | 19 | Hospital of origin | Culture (blood and bone marrow) | Immy alpha, immy GM, Miravista |  |
| 12 | Male, 45 |  | No | 23 | Hospital of origin | Culture (bone marrow) and histopathology (mediastinal  lymphadenopathy) | Immy GM and Miravista |  |
| 13 | Male, 36 | 9 | No | 92 | Bone marrow biopsy histopathology | Culture (blood and  lymphadenopathy biopsy) | Immy GM and Miravista | T cell NHL biopsy skin and bone  marrow |
| 14 | Male, 28 | 0 | Yes | 56 | Blood culture | - | Immy GM |  |
| 15 | Male, 27 | 2 | No | 92 | Culture (blood and lymphadenopathy) and lymphadenopathy biopsy  histopathology | - | Immy GM |  |
| 16 | Male, 33 | 80 | No | 5 | Culture (blood) | - | Immy alpha and Immy GM |  |
| 17 | Male, 36 | 138 | Yes | 8 | *Mycobacterium bovis* was identified  in blood and lymphadenopathy biopsy cultures | - | Miravista |  |
| 18 | Male, 33 | 0 | Yes | 34 | *Mycobacterium bovis* was identified in blood and bone marrow cultures; histopathology (bone marrow) | - | Immy GM | CMV colitis and HPV anal lesion by histopathology |
| 19 | Female,33 | 2 | Yes | 8 | Histopathology (granulomatous  hepatitis) and in Hospital of origin | - | Immy GM and Miravista |  |
| 20 | Female, 29 | 106 | No | 2 | Hospital of origin | - | Immy Alpha |  |
| 21 | Male, 21 | 0 | No | 77 | Hospital of origin | - | Immy GM |  |
| 22 | Male, 41 | 2 | No | 20 | Hospital of origin | - | Miravista |  |
| 23 | Male, 43 | 190 | No | 8 | Hospital of origin | - | Immy alpha |  |
| 24 | Male, 53 | 0 | Yes | NA | Hospital of origin | - | Immy GM |  |
| 25 | Male, 24 | 3 | Yes | 1 | Hospital of origin | - | Miravista | MAC |

**S2 Table.** Characteristics of the twenty-five PWH coinfected with histoplasmosis and tuberculosis (ARV=antiretroviral treatment; NA= not available; CMV=Cytomegalovirus; HPV=Human papilloma virus; MAC= *Mycobacterium avium* complex).
